# Targeting PKCθ in alloreactivity and graft-versus-host-disease: unanswered questions and therapeutic potential

**DOI:** 10.3389/fimmu.2012.00259

**Published:** 2012-08-14

**Authors:** Crystina C. Bronk, Xue-Zhong Yu, Amer A. Beg

**Affiliations:** ^1^Department of Immunology, Moffitt Cancer CenterTampa, FL, USA; ^2^Cancer Biology PhD Program, University of South FloridaTampa, FL, USA; ^3^Department of Oncologic Sciences, University of South FloridaTampa, FL, USA

**Keywords:** alloreactivity, graft-versus-host-disease, protein kinase C, T cell activation, anti-tumor effect

## Abstract

Protein kinase C isoform θ (PKCθ) is a key modulator of TCR signaling and mediates activation of NF-κB, NF-AT, and AP-1 transcription factors. Although *in vitro* studies of PKCθ^-/-^ T cells have shown impaired activation responses, *in vivo* studies indicate that PKCθ requirement is not universal. While PKCθ is important in induction of experimentally induced autoimmune diseases in mice and generation of Th2 responses, it is not essential for induction of T cell proliferative and cytotoxic responses against influenza virus, LCMV, and vaccinia virus. The context-specific involvement of PKCθ in T cell responses suggests that inhibition of PKCθ may be beneficial in some but not all situations. In the bone marrow transplantation (BMT) setting, we have shown that graft-versus-host-disease (GVHD) cannot be induced in the absence of PKCθ. However, graft-versus-leukemia effects and T cell ability to clear virus infection remains intact. Therefore, PKCθ is a potential therapeutic target in BMT, inhibition of which may prevent GVHD while retaining anti-tumor and anti-infection responses.

Studies of PKCθ^-/-^ mice have shown normal T cell development but greatly impaired *in vitro* proliferative responses (Sun et al., 2000; Pfeifhofer et al., 2003; Grumont et al., 2004). Several studies have shown that PKCθ exerts functions leading to survival of T cells and activation versus tolerance outcomes *in vivo* (Berg-Brown et al., 2004; Barouch-Bentov et al., 2005). In addition, PKCθ is crucial for induction of experimentally induced autoimmune diseases in mice, including encephalomyelitis, arthritis, and myocarditis (Salek-Ardakani et al., 2005; Anderson et al., 2006; Healy et al., 2006; Marsland et al., 2007). It has also been shown that T cells cannot mount Th2 responses in the absence of PKCθ, while induction of Th1 responses against *Leishmania major* are not substantially impacted (Marsland et al., 2004). PKCθ^-/-^ mice can also induce robust infection clearing CD8 T cell responses against multiple viruses including influenza virus, LCMV, and vaccinia virus (Marsland et al., 2004). Although *in vitro* proliferation of PKCθ^-/-^ T cells is typically impaired, why *in vivo* responses to infectious agents remain intact is not well understood. It is thought, however, that stimulation of innate immunity by these infectious agents bypasses the requirement for PKCθ *in vivo* (Marsland et al., 2005, 2007; Marsland and Kopf, 2008). Precisely how this is accomplished and which molecular pathways are involved in PKCθ-independent T cell activation is not clear. The context-specific requirement of PKCθ in T cell activation suggests that inhibition of PKCθ may be beneficial in some but not all situations. Thus, inhibiting PKCθ may be therapeutically beneficial, but these specific circumstances need to be identified.

## ESSENTIAL ROLE OF PKCθ IN ALLOREACTIVITY AND GRAFT-VERSUS-HOST-DISEASE

Alloreactivity is initiated by T cells that specifically recognize mismatched (non-self) MHC/peptide complexes. Graft-versus-host-disease (GVHD) is a potentially lethal complication of allogeneic bone marrow transplantation (BMT) in which alloreactive T cells from the donor are activated by mismatched major and/or minor histocompatibility complex antigens of the recipient. Although side-effects of BMT are severe, for many cancer patients this represents a last line of hope to remove residual tumor cells, as the alloantigen response mediating GVHD can also promote donor T cells to exert graft-versus-leukemia (GVL) effects (Shlomchik, 2007; Welniak et al., 2007). In this therapeutic BMT procedure, GVHD is the major complication as it leads to high morbidity and mortality of patients (Appelbaum, 2001; Shlomchik, 2007). To date, no clinical strategy has been established that can selectively prevent GVHD while preserving the GVL effect.

Given the differential requirement for PKCθ in distinct T cell-mediated responses, we investigated a potential role for PKCθ in the alloreactive responses of GVHD and GVL (Valenzuela et al., 2009). To investigate the necessity of PKCθ, we used an acute model of GVHD with the donor and recipient mismatched for both major and minor histocompatibility complex antigens (Liang et al., 2007). CD4 and CD8 T cells from wild-type (WT), PKCθ^-/-^ or NF-κB p50^-/-^cRel^-/-^ mice were transferred together with WT T cell-depleted (TCD) BM cells into lethally irradiated MHC mismatched recipients. p50^-/-^cRel^-/-^ T cells (Zheng et al., 2003) were used to investigate a potential requirement for NF-κB in this setting. As expected, recipients of WT T cells showed typical signs of GVHD with more than 70% mortality within 60 days after BMT. In a poignant divergence, the mice receiving PKCθ^-/-^ or p50^-/-^cRel^-/-^ T cells showed little evidence of GVHD and survived through the duration of the experiment. Additionally, histologic analysis of the small intestine (a major site of GVHD-induced tissue destruction) was normal in mice receiving PKCθ^-/-^ T cells, while their WT counterparts showed glandular destruction, lymphocytic infiltrate, and loss of mucosa. Although a clear mechanism remains elusive, additional studies indicated that GVHD does not occur in the absence of PKCθ because these T cells have impaired proliferation and increased apoptosis.

## PRESERVATION OF ANTI-VIRUS AND GVL RESPONSES BY T CELLS LACKING PKCθ

Allogeneic BMT results in increased risk of life threatening infections due to conditioning regimens these patients must undergo. Previous studies showed that responses to bacterial and viral agents remain intact in the absence of PKCθ (Marsland et al., 2005; Marsland and Kopf, 2008); yet, such studies have not been performed in a post-BMT setting. Because CMV is one of the most prevalent viruses carried by humans, it represents a major threat to BMT patients (Meyers et al., 1986). To recapitulate this disease, we used an MCMV infection model (Hossain et al., 2007, 2008). Importantly, both anti-MCMV T cell response and virus clearance was comparable between WT and PKCθ^-/-^ T cell transplanted mice (Valenzuela et al., 2009). Therefore, PKCθ is dispensable for a successful anti-MCMV response post-BMT.

The purpose of BMT in the cancer treatment settings is for donor T cells to be able to target malignant cells. Therefore, it is imperative that PKCθ^-/-^ T cells retain this GVL activity. To this end, we performed studies (Valenzuela et al., 2009) using the A20 B cell lymphoma line (Liang et al., 2007). Different numbers of WT and PKCθ^-/-^ T cells were transplanted, and evaluated for their ability to induce GVHD and mediate GVL activity. In the absence of T cells, all recipients died from lymphoma growth. All doses of WT T cells induced moderate to severe GVHD but little or no lymphoma growth. In contrast, all recipients of PKCθ^-/-^ T cells survived through the duration of the experiment with mild body weight loss and low GVHD. Furthermore, all the recipients of high PKCθ^-/-^ T cell numbers and most recipients of low or intermediate T cell numbers remained largely free of tumor. This result led us to the conclusion that PKCθ plays a substantial role in the induction of GVHD, but is not essential for GVL (Valenzuela et al., 2009). Importantly, these results indicate that pharmacological PKCθ targeting may impair GVHD without significantly impacting GVL responses.

## KEY UNRESOLVED ISSUES IN PKCθ FUNCTION

Previous studies and our findings indicate relatively normal responsiveness of PKCθ^-/-^ cells to infectious agents as well as high affinity antigenic stimulation (e.g., OVA) *in vivo* (Valenzuela et al., 2009). However, PKCθ^-/-^ T cell alloreactivity and GVHD-inducing ability is severely impaired, likely due to reduced proliferation and survival in recipient mice (Valenzuela et al., 2009). This fundamental difference in the requirement of PKCθ in various settings is a key unanswered question, and is central for understanding how detrimental and beneficial functions of T cells in BMT can be separated. The specific inability of PKCθ^-/-^ T cells to induce GVHD can be due to several mutually non-exclusive reasons. First, the conditioning regimen used for BMT may play an important role. Thus, lethal irradiation prior to BMT severely depletes recipient APC required for donor T cell activation. It is possible that reduction in APC impacts PKCθ^-/-^ T cell responses more severely than WT T cells. Interestingly, allograft survival in heart transplantation models showed a relatively small requirement for PKCθ in transplant rejection (Manicassamy et al., 2008; Gruber et al., 2009), likely due to presence of compensatory functions of PKCα (Gruber et al., 2009). Therefore, it is possible that impaired alloreactivity in the absence of PKCθ is more pronounced in the BMT setting. Second, it is possible that defects in CD4 and CD8 T cell migration (Letschka et al., 2008) contribute to lack of GVHD induction in the absence of PKCθ. Thus, impaired migration of PKCθ^-/-^ T cells to GVHD target organs such as the gut, lungs, and skin may be responsible for reduced GVHD. Third, the function of PKCθ in alloreactivity may not be limited to effector T cell responses. Previous studies investigating a role for PKCθ in Tregs suggest that PKCθ function in Treg may also be important in alloreactivity (Zanin-Zhorov et al., 2010). While PKCθ localizes to the immune synapse (IS) in effector T cell, PKCθ is sequestered in a distal complex away from the IS in Treg (Zanin-Zhorov et al., 2010). As such, PKCθ is responsible for mediating a negative effect on the suppressive function of Treg. Consequently, PKCθ inhibition enhances Treg function leading to protection from inflammatory colitis in mice (Zanin-Zhorov et al., 2010). A caveat worth mentioning here is that while PKCθ inhibition leads to enhanced Treg function, PKCθ absence does not have the same effect (Gupta et al., 2008). The underlying reason for this is not completely clear (Zanin-Zhorov et al., 2011).

The easiest albeit simplistic way to understand why PKCθ absence does not impact anti-infection and anti-tumor responses is to consider a role for functionally redundant pathways. As mentioned above, PKCθ is involved in regulating activation of NF-κB, AP-1, and NF-AT. Studies by Marsland and colleagues have shown that microbial stimulation through pattern recognition receptors (PRR) can induce NF-κB activation in PKCθ^-/-^ T cells (Marsland et al., 2005, 2007; Marsland and Kopf, 2008). Thus, PRR may play a key functionally redundant role with PKCθ during infection with microbial agents. In contrast, why anti-tumor responses are only slightly reduced in the absence of PKCθ is more difficult to understand. BMT is primarily used for leukemia treatment. Leukemic cells, in particular B lymphocytes, have naturally high expression of MHC and co-stimulatory molecules, reflecting the natural function of these lineages in antigen presentation. In the above-mentioned study (Valenzuela et al., 2009), A20 B cell lymphoma cells were used as tumor targets. Whether PKCθ^-/-^ T cells are specifically (or only) able to eradicate leukemic tumors can be directly tested by determining PKCθ requirement in eradication of non-leukemic tumors. Mechanistically, one possibility is that functionally redundant pathways are strongly activated in PKCθ^-/-^ T cells by A20 and potentially other leukemic tumors. Furthermore, leukemic tumors may represent better targets for PKCθ^-/-^ T cells than epithelial cells targeted during GVHD. Regardless of precise mechanisms, it is likely that both responses to infectious agents and leukemic tumors are maintained in the absence of PKCθ through functionally redundant pathways. A recent study identified a novel role for PKCθ as a transcriptional co-activator capable of interacting with promoters of several immune function genes (Sutcliffe et al., 2011). How this function impacts alloreactivity and other known functions for PKCθ remains to be determined.

## INVESTIGATING THE EFFECT OF PKCθ INHIBITION IN GVHD AND GVL

The above-mentioned studies provide strong rationale for targeting PKCθ in a BMT settings (Valenzuela et al., 2009). The PKC family has been implicated in tumor cell proliferation, survival, invasion, metastasis, and tumor angiogenesis. Therefore, targeting PKC isotypes may present an attractive target for novel anticancer therapies. Consequently, several inhibitors of PKC isoforms have been developed by different pharmaceutical companies. Although some selective PKCθ inhibitors have been reported (Cywin et al., 2007; Cole et al., 2008), their *in vivo* toxicity or efficacy remains to be determined. A major advance was achieved recently with AEB071 (developed by Novartis), a very potent and selective inhibitor of both novel and classical PKCs. Skvara et al. (2008) reported that treatment with AEB071 significantly improved symptoms of patients with severe psoriasis. Another potentially important PKC inhibitor is enzastaurin (Ly317615, from Eli Lilly and Company; Baier and Wagner, 2009). Enzastaurin was identified as an inhibitor of the PKCβ isoform, but it also impacts the AKT pathway (Graff et al., 2005); importantly, enzastaurin inhibits the PKCθ isoform approximately fivefold more potently than the beta isoform (Graff et al., 2005). *In vivo* studies indicate that enzastaurin is very well tolerated with a favorable safety profile, allowing it to be dosed for extended durations (Herbst et al., 2007). Furthermore, enzastaurin has anti-proliferative and pro-apoptotic activity in solid tumors as well as hematological malignancies, including leukemia and lymphoma (Herbst et al., 2007). Thus, while not exclusively specific for PKCθ, enzastaurin has the major advantage of being well tolerated *in vivo* and the added benefit of anti-tumor effects. Hence, PKCθ inhibition by enzastaurin may prevent GVHD while preserving GVL responses, which will act cooperatively with the direct anti-tumor effects of enzastaurin. We expect future studies to define more potent and specific inhibitors of PKCθ and consequently help move this important therapeutic target to the clinical arena.

## Conflict of Interest Statement

The authors declare that the research was conducted in the absence of any commercial or financial relationships that could be construed as a potential conflict of interest.
